# Near-infrared fluorescence imaging of hepatocellular carcinoma cells regulated by β-catenin signaling pathway

**DOI:** 10.3389/fonc.2023.1140256

**Published:** 2023-03-29

**Authors:** Jian Song, Tingting Ren, Yanheng Duan, Haitao Guo, Gang Wang, Yu Gan, Mengcai Bai, Xiaotian Dong, Zheng Zhao, Jiaze An

**Affiliations:** ^1^ Department of Hepatobiliary Surgery, Xijing Hospital, Fourth Military Medical University, Xi’an, China; ^2^ State Key Laboratory of Cancer Biology and Experimental Teaching Center of Basic Medicine, Fourth Military Medical University, Xi’an, China; ^3^ State Key Laboratory of Cancer Biology and Department of Physiology and Pathophysiology, Fourth Military Medical University, Xi’an, China; ^4^ Third Department of Medical Oncology, Shaanxi Provincial Cancer Hospital, Xi’an, China

**Keywords:** near-infrared fluorescence imaging, MHI-148, β-catenin signaling pathway, HCC (hepatic cellular carcinoma), ICG = indocyanine green

## Abstract

**Background:**

Near-infrared fluorescence (NIRF) imaging has recently emerged as a promising tool for noninvasive cancer imaging. However, lack of tumor sensitivity and specificity restricts the application of NIRF dyes in surgical navigation.

**Methods:**

Herein, we investigated the imaging features of NIRF dye MHI-148 and indocyanine green (ICG) in live cell imaging and xenograft nude mice models. TCGA dataset analysis and immunohistochemistry were conducted to investigate the expression of OATPs or ABCGs in hepatocellular carcinoma (HCC) tissues. OATPs or ABCGs were knocked down and overexpressed in HCC cells using transient transfection by siRNA and plasmids or stable transfection by lentivirus. Further, qRT-PCR ,Western blotting and the use of agonists or inhibitors targeting β-catenin signaling pathway were applied to explore its important role in regulation of OATP2B1 and ABCG2 expression.

**Results:**

Here we demonstrated that NIRF dye MHI-148 was biocompatible as indocyanine green (ICG) but with higher imaging intensity and preferential uptake and retention in hepatocellular carcinoma (HCC) cells and tissues. Moreover, our data indicated that membrane transporters OATP2B1 and ABCG2, which regulated by β-catenin signaling pathway, mediated tumor-specific accumulation and retention of MHI-148 in HCC cells. In addition, the treatment with β-catenin inhibitor significantly enhanced the accumulation of MHI-148 in HCC tissues and improved the efficacy of tumor imaging with MHI-148 *in vivo*.

**Conclusions:**

Our study uncovers a mechanism that links the distribution and expression of the membrane transporters OATP2B1 and ABCG2 to the tumor-specific accumulation of MHI-148, and provides evidence supporting a regulating role of the β-catenin signaling pathway in OATP2B1 and ABCG2- induced retention of MHI-148 inHCC tissues, and strategy targeting key components of MHI-148 transport machinery may be a potential approach to improve HCC imaging.

## 1 Introduction

Hepatocellular carcinoma (HCC), which represents approximately 90% of primary liver cancer, is currently the second leading cause of cancer-related death globally with an age-standardized 5-year survival rate of 20% (1, 2). As a main therapeutic strategy to achieve long-term survival of HCC patients, hepatectomy is required for removal of primary and disseminated tumors and maximum preservation of normal liver tissue (3, 4). However, one of the most difficult problem in liver tumor resection is to accurately locate the positive margins and remove all existing tumors (5).

Near infrared fluorescence (NIRF) imaging, as a real-time and highly sensitive imaging technique, has been extensively studied in surgical navigation and animal experiments (6, 7). The most representative NIRF dye indocyanine green (ICG) is widely used in measurement of liver clearance or hepatectomy in hepatocellular carcinoma (HCC), which has already been approved by the Food and Drug Administration (FDA) and the European Medicines Agency (EMEA) (8). However, lack of tumor sensitivity and specificity leads to a high false positive rate in the intraoperative detection of tumor nodules (9). Therefore, these limitations restrict the application of ICG in tumor localization. Since numerous HCC related biomarkers have been reported (10, 11), molecular targets and ligand binding played a crucial role in developing disease-targeted molecular imaging probes. Cheng et al. has been synthesized a GPC3 targeted ICG probe which demonstrates specific HCC targeted imaging capability, and can clearly delineate the tumor (12). Tian et al. also successfully constructed an active tumor-selective ICG probe, ICG/MSNs-RGD, using MSNs with a high ICG-loading ability and high α_v_β_3_ receptor targeting specificity, which demonstrated precise tumor margin delineation during liver cancer surgery (13).

Although a large number of near infrared probe has been developed to improve the accuracy of tumor detection and the signal-to-background ratio (14–17), clinical transformation still faces great challenges due to the lack of sufficient biocompatibility and tumor biological affinity. Recent studies have reported a specific class of near-infrared heptamethine carbocyanine dye with tumor-targeting properties, indicating that NIRF dye MHI-148 can be used for effective imaging with good stability and specificity in different types of tumor cell lines and tumor xenografts, such as kidney cancer, prostate cancer and lung cancer (18–20). Thomas et al. have also used MHI-148 as a tumor-targeting agent conjugated to nanoparticle for breast cancer treatment (21). Although these studies take advantage of the tumor-specific accumulation of MHI-148 for tumor detection and therapy, there is yet limited understanding of the mechanisms involved in the uptake and retention of MHI-148 in HCC cells.

Absorption, distribution, and excretion of endogenous and exogenous compounds are involved in transport of substances across plasma membrane, most of which are mediated by drug transporters (22). These transporters are key determinants of drug accumulation within cells, whose activities are often directly related with clinical efficacy, toxicity, and interactions between agents. These transporters are generally divided into solute carrier (SLC) family and ATP-binding cassette (ABC) family. Most of SLC transporters belong to influx transporters which mediate movement of solutes from extracellular milieu into cells. ABC transporters belong to efflux transporters that export drugs out of cells using ATP as driving energy (23). Recently, several evidences have revealed that a group of SLCs called organic anion-transporting polypeptides (OATPs) are involved in the entry of NIRF dyes into tumor cells. Takeaki et al. have reported that the preserved portal uptake of ICG in differentiated HCC cells by OATP8 with concomitant biliary excretion disorders causes accumulation of ICG in the cancerous tissues after preoperative intravenous administration (24). Hsiao et al. have verified that ICG could be mainly ingested by OATP1B1 and OATP1B3 in HT-1080 fibrosarcoma cells and HT-29 colon cancer cells (25, 26). Wu et al. have demonstrated that OATP1B3 mediates the uptake of NIRF dye by cancer cells and tumor xenografts (27). Although it has been suggested that the SLC transporters may be an important regulator of the tumor-specific absorption and distribution of NIRF dye MHI-148, there is yet limited understanding of whether ABC transporters is involved in the efflux of MHI-148 in HCC cells, and what the exact mechanisms underlying the effect of membrane transporters on tumor-targeting properties of MHI-148 are.

In the present study, we first systematically investigated the application of NIRF dye MHI-148 in imaging of HCC tissues. Moreover, the molecular mechanisms underlying membrane transporters-mediated tumor-specific accumulation and retention of MHI-148 in HCC cell were intensely explored. Our study provides the supporting evidence for the novel strategy targeting key components of MHI-148 transport machinery in clinical imaging of HCC tissues.

## 2 Materials and methods

### 2.1 Antibodies and reagents

Antibodies and their working concentrations used in this study were listed in **Supplementary Table 1**. CHIR99021 (Cat# HY-10182) and XAV939 (Cat# HY-15147) were purchased from MCE (MCE, USA). Prodigiosin (Cat# P274778) was purchased from Aladdin (Aladdin, China). MHI-148 (Cat# M-21002) and ICG (Cat# C-34500) were purchased from HEOWNS (HEOWNS, China). Hoechst 33342 (Cat# M5112) was purchased from ABMOLE (ABMOLE, USA). And the chemical structure of ICG and MHI-148 is shown in **Supplementary Figure 1**.

### 2.2 Cell culture

Human HCC cell lines HLF and HLE were obtained from Japanese Collection of Research Bioresources (Osaka, Japan). SNU-368, SNU-398 and SNU-739 were obtained from the Korean Cell Line Bank (KCLB, Seoul, Korea). Huh-7 and 293T cells were purchased from Shanghai Cell Bank of the Chinese Academy of Sciences (Shanghai, China). Human normal liver epithelial cell line THLE-2 was purchased from SHXY Bio (Cat#C638). All cell lines were authenticated using short tandem repeat DNA testing by the FMMU Center for DNA Typing and cultured in DMEM or RPMI1640 (Gibco, Grand Island, NY) supplemented with 10% fetal bovine serum (Hyclone, Logan, UT) and kept at 37°C with 5% CO_2_.

### 2.3 Tissue and public dataset collection

Tissue samples from surgical HCC patients were obtained in Xijing Hospital affiliated with Fourth Military Medical University, Xi’an, China. The study was approved by the Ethics Committee of the Fourth Military Medical University and written informed consent was obtained from all participants. The characteristics of HCC patients were summarized in **Supplementary Table 2**. Moreover, TCGA HCC dataset was retrieved from the Genomic Data Commons Portal. The data were analyzed using GraphPad Prism v.8.0. Incomplete data, missing expression values and survival were eliminated from the analysis, and only primary tumors were considered.

### 2.4 Cell viability assay

Cell viability was examined by the MTS assay (Promega Corporation, G3581). SNU-739 cells were seeded in 96-well plates at 1 × 10^4^ cells/well and cultured at 37°C with 5% CO_2_ for 24h. MTS reagent was added to each well and the plate was subsequently incubated for additional 2h at 37°C. The microplates were read in a spectrophotometer at a wavelength of 490 nm. Each sample was analyzed in triplicate.

### 2.5 Quantitative RT-PCR, western blotting and immunohistochemistry

Total proteins were extracted with RIPA buffer (Beyotime Institute of Biotechnology, Shanghai). Whole cytoplasmic proteins and nuclear proteins were extracted using Nuclear and Cytoplasmic Protein Extraction Kit (Beyotime, Shanghai). RNA extraction, complementary DNA synthesis, qRT-PCR reactions and Western blotting were performed as previously described (28). Primer sequences used were provided in **Supplementary Table 3**. Processing of HCC tissues for immunohistochemistry (IHC) and evaluation of IHC staining were carried out as previously described (29).

### 2.6 Knockdown and forced expression of target genes

Small interference RNA (siRNA) targeting SLC transporters and ABC transporters, and control siRNA (Tsingke Biotechnology, Beijing) were transfected into HCC cell lines using the transfection reagent lipofectamine 2000 (Thermo Fisher Scientific, MA, USA) according to the manufacturer’s protocols. Sequences of siRNA used in the study were listed in **Supplementary Table 3**.

pIRES2-EGFP vector was used to express HNF4α (Tsingke Biotechnology, Beijing). The lentiviral pLVX-puro vector was used to express OATP2B1, and the lentiviral pSLenti-SFH-EGFP-P2A-Puro-CMV-MCS-3xFLAG-WPRE vector was used to express ABCG2 (Obio Technology, Shanghai). Lentiviral pLKO.1 vector was used to express short hairpin RNA (shRNA) directed against OATP2B1 (sh-OATP2B1), ABCG2 (sh-ABCG2) or a non-silencing scrambled control sequence. The sequences were listed in **Supplementary Table 3**. 293T cells were transfected with the constructs according to the manufacturer’s protocol, and recombinant lentiviruses were typically generated within 48-72 hours. HCC cells (8×10^5^ cells/well) were seeded in 6-well plates and incubated with lentiviruses for 24 hours at 37°C and replaced with fresh medium for additional 72 hours. Subsequently, the cells were selected by treatment with 1.5 µg mL^-1^ puromycin (Cayman, USA) for 4 days.

### 2.7 NIRF imaging *in vitro*

HCC cells and normal liver epithelial cells were seeded in dishes for 24 hours and then treated with ICG (Ex/Em,750-810/840 nm) or MHI-148 (Ex/Em,760-780/820-860 nm) which was dissolved in DMSO with its volume being less than 0.1% (v/v) in the cell medium. Cells were incubated at 37°C and washed twice with PBS to remove excess dyes. Cell nuclei was stained with Hoechst 33342. Uptake of the NIRF dyes was analyzed by a NIR fluorescence microscopy (Olympus 1×71, Olympus, Melville, NY) equipped with a 75 W Xenon lamp and an indocyanine green filter cube (excitation/emission, 750-800/820-860 nm: exposure time, 30 s). Cellular NIRF intensity was quantified by Image J software (NIH, Bethesda, MD).

### 2.8 Nude mice xenograft model and NIRF imaging *in vivo*

Male BALB/c nude mice, 6–8 weeks of age, were purchased from Vital River (Beijing, China), bred in an SPF-level barrier environment at the Laboratory Animal Center within the Fourth Military Medical University (FMMU), and randomly divided into groups (5 mice/group). To establish subcutaneous tumor xenograft models, 2 × 10^6^ HCC cells were implanted subcutaneously into the left shoulder of nude mice. Two weeks later, the mice bearing tumors were injected with ICG or MHI-148 (0.75 μmol kg^-1^) through tail vein. After 0.5, 1, 2, 4, 8, 24 and 48h, NIRF intensity was measured at the tumor site and within major organs (including heart, liver, spleen, lung, intestines and kidney) by a Lumina II Small Animal Optical Imaging System (Caliper Life Sciences, Hopkinton, MA, USA) with fluorescent field sets (excitation/emission, 760/850 nm). After the tumor volume reached approximately 50 mm^3^, mice were treated by i.p. injection with vehicle or Prodigiosin at 5 mg kg-1 twice weekly for 1 week, then MHI-148 was injected through the tail vein. And NIRF intensity was measured at the tumor site by a Lumina II Small Animal Optical Imaging System (Caliper Life Sciences, Hopkinton, MA, USA) with fluorescent field sets (excitation/emission, 760/850 nm). The camera settings included maximal gain, 2 × 2 binning and an exposure time of 5 s. Mice were maintained under isoflurane during imaging (RC2 Rodent gas anesthesia machine). All animal experiments were approved (8 October 2020) by the animal welfare ethics committee of FMMU (IACUC-20201008).

### 2.9 Biosafety analysis *in vivo*

Healthy mice (n=5) were injected intravenously with ICG or MHI-148 at a dosage of 0.75 μmol kg^-1^. Following these injections, the mice were weighed at various time points from 0~30 days. Meanwhile, animal behaviors were also carefully recorded. At day7 and 30 after injection, the mice were anaesthetized and blood samples were collected for blood biochemistry test (Chengdu Lilai Biotechnology Co., Ltd). The mice injected with saline and DMSO were used as control. Subsequently, the main organs of the mice (heart, liver, spleen, intestines, lung and kidney) were harvested and fixed using 4% paraformaldehyde. Tissue samples were then embedded in paraffin, sliced (5μM) and stained using haematoxylin and eosin. All of the obtained biopsy samples were imaged using an optical microscope (Leica).

### 2.10 Statistical analysis

SPSS 17.0 software (SPSS. Inc., USA) was utilized to analyze the data. A *P*-Value <0.05 was considered statistically significant. Data shown as the mean ± SD from three independent experiments, where appropriate. No statistical methods were used for sample size selection. The significance of the results was determined employing two-tailed unpaired Student’s *t*-test (when comparing two groups) or one-way analysis of variance (ANOVA) for multiple comparisons (when more than two groups were compared).

## 3 Results

### 3.1 Near-infrared fluorescence imaging of HCC cells by NIRF dyes MHI-148 and ICG *in vitro* and *in vivo*

**Figure 1:**
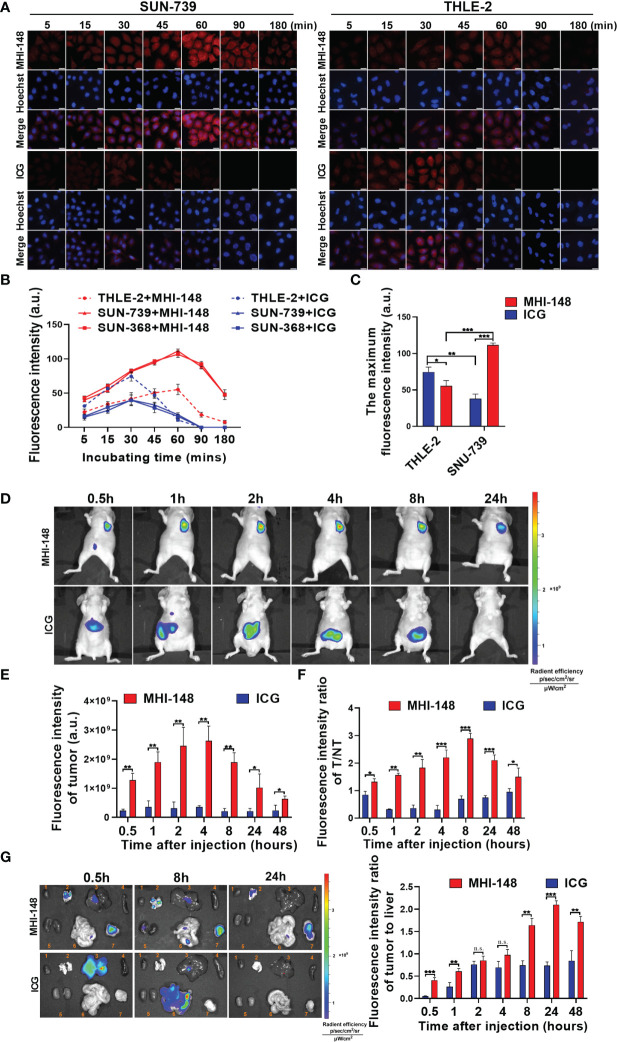
Near-infrared fluorescence imaging of HCC cells by NIRF dyes MHI-148 and ICG *in vitro* and *in vivo*. **(A)** Representative images of cells staining with NIRF dyes in SNU-739 cells and THLE-2 cells. Cells were treated with 10μM MHI-148 or ICG for different time as indicated. Scale bar, 20 μm. **(B)** Quantification of NIRF intensity in SNU-739, SNU-368 cells and THLE-2 cells. **(C)** The maximum NIRF intensity was compared in SNU-739 cells and THLE-2 cells, respectively. **(D)** Time-dependent NIRF intensity images of nude mice bearing subcutaneous tumors xenografts of SNU-739 cells after receiving an intravenous injection of MHI-148 or ICG. **(E)** Quantification of NIRF intensity in tumor. **(F)** Quantification of NIRF intensity ratios of tumor tissues to normal tissues against time. **(G)** Tumor tissues and normal tissues were dissected and subjected to ex vivo imaging at 0.5h, 8h and 24h after injection (left) and quantification of NIRF intensity ratios of tumor to liver (right). The organs numbered from 1 to 7 in the pictures are heart, lung, liver, spleen, kidney, intestine and tumor tissue. Data shown are the mean ± SD from three independent experiments, where appropriate. *P < 0.05; ** P < 0.01; *** P < 0.001.

Firstly, the specific uptake of NIRF dyes MHI-148 and ICG was assessed in HCC cells (SNU-739, SNU-368) and human liver epithelial cells (THLE-2). Dosage and time-dependent dyes clearance from HCC cells is shown in (**Supplementary Figure 2A**). A significantly higher uptake of MHI-148 was observed by the HCC cells, compared with ICG at all dye concentrations. However, it is difficult to observe the change of fluorescence intensity with low concentration of dyes (0.1μM and 1μM), and high concentration of dyes is toxic to cells (**Supplementary Figure 2B**). So, we used an appropriate concentration (10μM) for *in vitro* studies. As shown in **Figures 1A, B** and **Supplementary Figure 2C**, significant MHI-148 uptake was observed in SNU-739 and SNU-368 rather than in THLE-2 cells, and the peak intensity was reached at 60 minutes after treatment. Near background uptake of ICG was observed in SNU-739 and SNU-368. In contrast, ICG was absorbed rapidly in THLE-2 and the signal peak appeared at 30 minutes after treatment. Then a sharp drop in fluorescence signals was observed, indicating rapid excretion of ICG. By comparing the maximum fluorescence intensity between the two dyes in SNU-739 cells or THLE-2 cells, our results showed that MHI-148 preferred to be absorbed by HCC cells and presented a higher fluorescence intensity, while ICG aggregated predominantly in liver epithelial cells (**Figure 1C**).

To investigate any *in vivo* toxicity, we selected a series of dosages of ICG/MHI-148 (0, 0.5, 0.75, 1, 1.5, 2 μmol kg^-1^) to screen the safe concentration *in vivo*. It was observed that the experimental mice with injection dose of higher than 0.75 μmol kg^-1^either died within 5 days or lost weight within one week after the tail vein injection as shown in **Supplementary Figure 2D**. And the 2 μmol kg^-1^ group died on the day after intravenous injection (Data not displayed). For the mice were treated with MHI-148 and ICG (0.75 μmol kg^-1^), there were no significant differences in body weights over a 30-d period (**Supplementary Figure 2E**). Also, no side effects on physical signs were found. For the blood biochemistry test, negligible fluctuations in 6 important hepatic, heart and kidney function biochemical indexes indicated the absence of acute or chronic toxicity (**Supplementary Figure 2F**). Haematoxylin and eosin-stained images indicated that structural patterns of major organs harvested on day 30 from mice in the experimental group were similar to those of the control group, with no signs of damage or other symptoms. (**Supplementary Figure 2G**) These data collectively suggest that MHI-148 and ICG can be biocompatible.

We then evaluated the uptake of MHI-148 and ICG *in vivo*. As shown in **Figure 1D**, MHI-148 was distributed mainly in tumor area, while high signals were detected in the abdomen of mice after ICG administration. Accumulation of NIRF dyes in tumor was quantified, and we found that MHI-148 presented a high fluorescence intensity which continued to increase and peaked at 4 hours after injection, whereas ICG had a consistently low signal in tumor (**Figure 1E**). Simultaneously, compared with the virtually unchanging tumor-to-normal tissue (T/NT) values for the ICG, the values for MHI-148 reached its peak at 8 hours after injection (**Figure 1F**). Moreover, the specific fluorescence signal of MHI-148 in tumor xenografts persisted for at least 3 days before gradually fading (data not shown). The fluorescence signal of tumor and normal tissues was also compared by ex vivo NIR imaging after injection of the NIRF dyes. As shown in **Figure 1G**, MHI-148 generated signals with widespread intensity in both tumor and non-tumor tissues at first, and then accumulated in tumor gradually. However, ICG only exhibited strong brightness in liver and the fluorescent signal was presented in the intestine with time, indicating that ICG was only absorbed by normal liver cells and metabolized in intestine. The fluorescence intensity ratio of MHI-148 in tumor to liver was increased significantly at 8 hours after injection, reaching over 2-fold higher than ICG, and lasting until 48 hours after injection.

### 3.2 OATP2B1 is involved in uptake of MHI-148 into HCC cells

**Figure 2:**
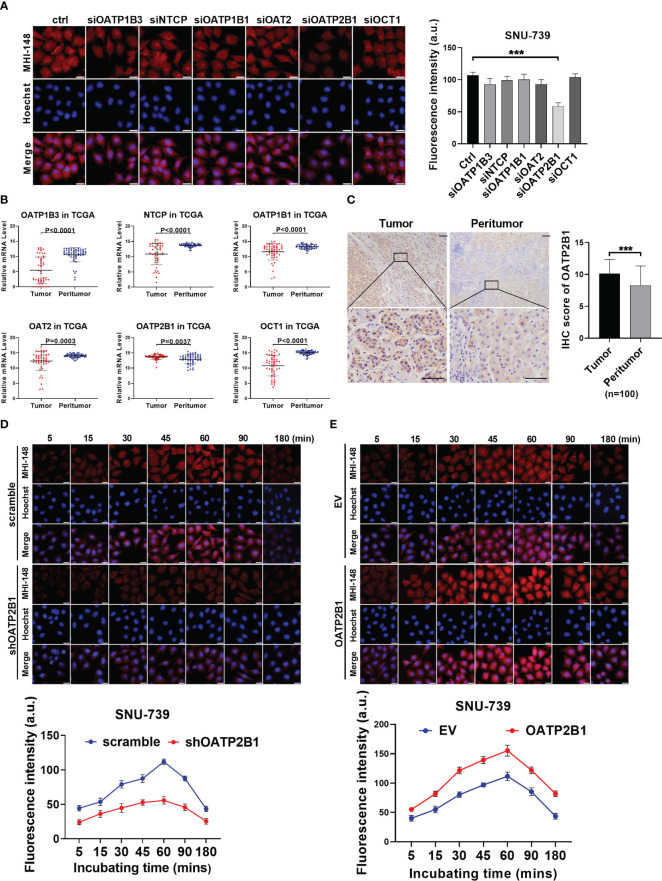
OATP2B1 is involved in uptake of MHI-148 into HCC cells *in vitro*. **(A)** Near-infrared fluorescence imaging of SNU-739 cells transfected with siRNAs against different SLC transporters and stained with MHI-148 for 60 minutes. **(B)** The relative mRNA expression levels of tumor and peritumor of SLC transporters were analyzed by transcriptome sequencing data from The Cancer Genome Atlas (TCGA). **(C)** Representative immunohistochemical (IHC) staining image (Left) and IHC score (Right) of OATP2B1 in 100 paired HCC tissues (tumor and peritumor). Data are expressed as mean ± SD. Scale bar, 50µm. **(D, E)** Determination of MHI-148 uptake in SNU-739 cells with knockdown or overexpression of OATP2B1. Data shown are the mean ± SD from three independent experiments, where appropriate. ***P < 0.001.

The super family of solute carrier (SLC) transports is well known to mediate the transport of endogenous and exogenous substances into cells in a highly substrate -dependent manner. To explore whether SLC transports participated in the uptake of MHI-148 into HCC cells, the fluorescence intensity of MHI-148 was assessed in SNU-739 after transfection of specific siRNAs against six kinds of SLC transporters which are mainly localized in hepatocytes (including OATP1B3, NTCP, OATP1B1, OAT2, OATP2B1 and OCT1). Our results showed that cells only with OATP2B1 knockdown exhibited a significant decrease of fluorescence intensity at 60 minutes after treatment, suggesting that OATP2B1 may play a critical role in uptake of MHI-148 into HCC cells (**Figure 2A** and **Supplementary Figure 3A**). Bioinformatic analysis based on public transcriptome sequencing data from The Cancer Genome Atlas (TCGA) database indicated that OATP2B1 was up-regulated in HCC tissues when compared with paired non-tumor tissues (**Figure 2B**), which was also demonstrated in cell lines by western Blotting (**Supplementary Figure 3B**). Immunohistochemical staining analysis in a large cohort of 100 HCC patients further confirmed the above-mentioned results (**Figure 2C**).

To directly investigate the functional role of OATP2B1 in the uptake of MHI-148 dye into HCC cells, cells with the relatively low or high expression of OATP2B1 were selected for the establishment of cell models with knockdown or forced expression of OATP2B1, respectively (**Supplementary Figure 3C**). Near-infrared fluorescence imaging analysis showed that the decreased expression of OATP2B1 was associated with a drop of MHI-148 fluorescence signal than controls (The maximum fluorescence intensity at 60 minutes respectively is 56 ± 5 and 112 ± 4 a.u.), whereas cells with overexpression of OATP2B1 had an enhanced fluorescence signal than controls (The maximum fluorescence intensity at 60 minutes respectively is 155 ± 9 and 111 ± 7 a.u.) (**Figures 2D, E** and **Supplementary Figures 3D, E**).

**Figure 3:**
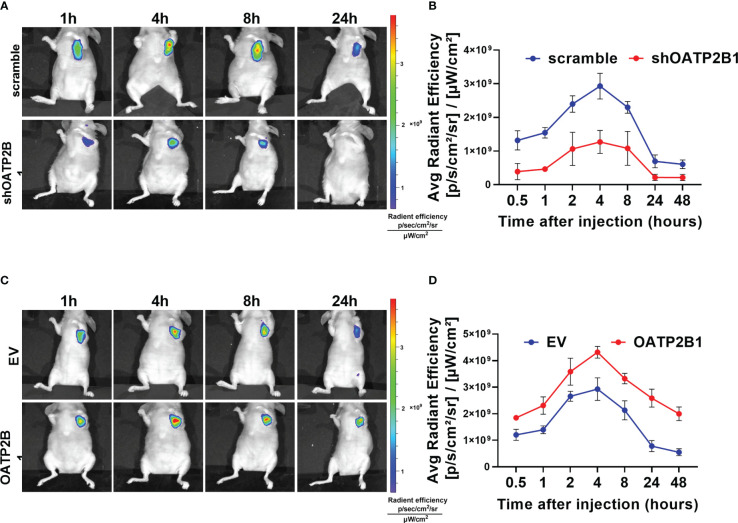
OATP2B1 is involved in uptake of MHI-148 in tumor xenografts. **(A)** Time-dependent intensity images of nude mice bearing subcutaneous tumors xenografts of SNU-739 cells with OATP2B1-knockdown (shOATP2B1) after receiving an intravenous injection of MHI-148 (0.75 μmol kg^-1^). **(B)** Quantification of MHI-148 fluorescence intensity in **(A)**. **(C)** Time-dependent intensity images of nude mice bearing subcutaneous tumors xenografts of SNU-739 cells with overexpressed OATP2B1 (OATP2B1) after receiving an intravenous injection of MHI-148 (0.75 μmol kg^-1^). **(D)** Quantification of MHI-148 fluorescence intensity in **(C)**. Data shown are the mean ± SD from three independent experiments, where appropriate.

Further, we examined the effect of OATP2B1 expression on the uptake of MHI-148 in tumor-bearing mice. As shown in **Figures 3A, B**, OATP2B1 knockdown significantly reduced the ability of tumor xenografts to take up and retain MHI-148 by up to 43% when compared with control group at 4h after the tail vein injection (The average radient efficiency respectively is 1.27 ± 0.3×10^9^ and 2.9 ± 0.4×10^9^ [p/s/cm^2^/sr]/[μW/cm^2^]). In contrast, overexpression of OATP2B1 not only increased the uptake rate of MHI-148, but also enhanced MHI-148 fluorescence intensity by about 30% relative to control group (The average radient efficiency respectively is 4.3 ± 0.2×10^9^and 2.8 ± 0.5×10^9^ [p/s/cm^2^/sr]/[μW/cm^2^]) (**Figures 3C, D**).

These results support the functional role of OATP2B1 in transporting MHI-148 into HCC cells and suggest that OATP2B1 may be a promising regulator in clinical application of MHI-148.

### 3.3 The efflux of MHI-148 from HCC cells is mainly mediated by ABCG2

**Figure 4:**
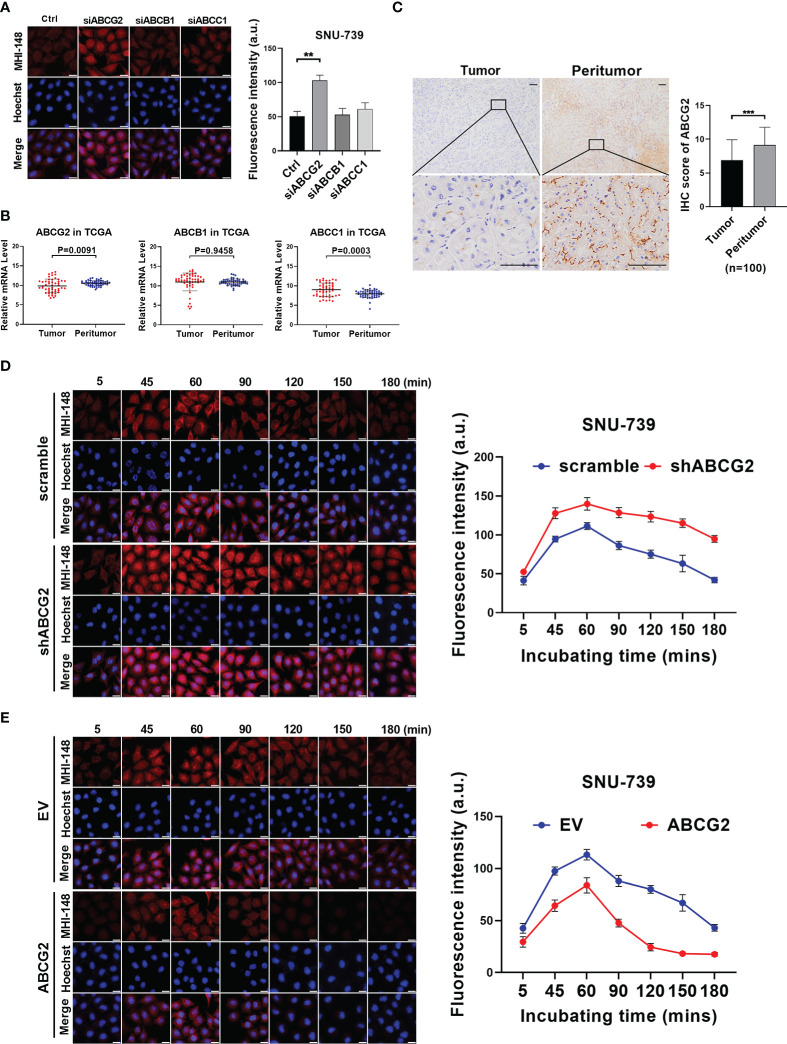
The efflux of MHI-148 from HCC cells is mainly mediated by ABCG2 *in vitro*. **(A)** Near-infrared fluorescence imaging of SNU-739 cells transfected with siRNAs against different ABC transporters and stained with MHI-148 for 180 minutes. **(B)** The relative mRNA expression levels of tumor and peritumor of ABC transporters were analyzed by transcriptome sequencing data from TCGA. **(C)** Representative IHC staining image (left) and IHC score (right) of ABCG2 in 100 paired HCC tissues (tumor and peritumor). Data are expressed as mean ± SD. Scale bar, 50µm. **(D, E)** Determination of MHI-148 retention in SNU-739 cells with knockdown or overexpression of ABCG2. Data shown are the mean ± SD from three independent experiments, where appropriate. **P < 0.01; *** P< 0.001.

The accumulation of NIRF dyes in cells was affected by both uptake and efflux, and the decreased efflux of dye from HCC cells resulted in the increased intracellular fluorescence signal. Previous studies have demonstrated that human ATP-binding cassette (ABC) transporters, a large group of membrane protein complexes, used the energy generated by ATP hydrolysis to expel exogenous substances from cells. Therefore, we explored whether ABC transporters mediated MHI-148 efflux from HCC cells. Fluorescence intensity of MHI-148 was assessed in SNU-739 cells after transfection of specific siRNAs against ABCs including ABCG2, ABCB1 and ABCC1, which were reported to be able to transport numerous diverse chemical substrates out of cells and were associated with drug efflux in HCC cells. Our results showed that cells only with ABCG2 knockdown exhibited a significant increase of fluorescence intensity at 180 minutes after injection, suggesting that ABCG2 may play a critical role in efflux of MHI-148 from HCC cells (**Figure 4A** and **Supplementary Figure 4A**). Bioinformatic analysis based on public transcriptome sequencing data from TCGA database indicated that ABCG2 was down-regulated in HCC tissues when compared with paired non-tumor tissues, which was also demonstrated in cell lines by Western Blotting (**Figure 4B** and **Supplementary Figure 4B**). Immunohistochemical staining analysis in a large cohort of 100 HCC patients further confirmed the above-mentioned results (**Figure 4C**).

We then investigated the role of ABCG2 in MHI-148 fluorescence imaging in HCC cells. Cells with the relatively low or high expression of ABCG2 were selected for the establishment of cell models with knockdown or forced expression of ABCG2, respectively (**Supplementary Figure 4C**). Near-infrared fluorescence imaging showed that the restrained expression of ABCG2 was associated with a slight increase of fluorescence signal in HCC cells incubated with MHI-148 for 60 minutes, but more remarkably, the brightness lasted longer than control (The maximum fluorescence intensity at 60 minutes respectively is 140 ± 8 and 110 ± 4 a.u.) (**Figure 4D** and **Supplementary Figure 4D**). Meanwhile, cells with overexpression of ABCG2 remarkably reduced the ability of HCC cells to retain MHI-148. The fluorescence signal sharply decreased after reaching the peak at 60 minutes after injection and continued to drop to the lowest at 150 minutes after injection than control (The maximum fluorescence intensity at 60 minutes respectively is 84 ± 7 and 113 ± 5 a.u.) (**Figure 4E** and **Supplementary Figure 4E**).

**Figure 5:**
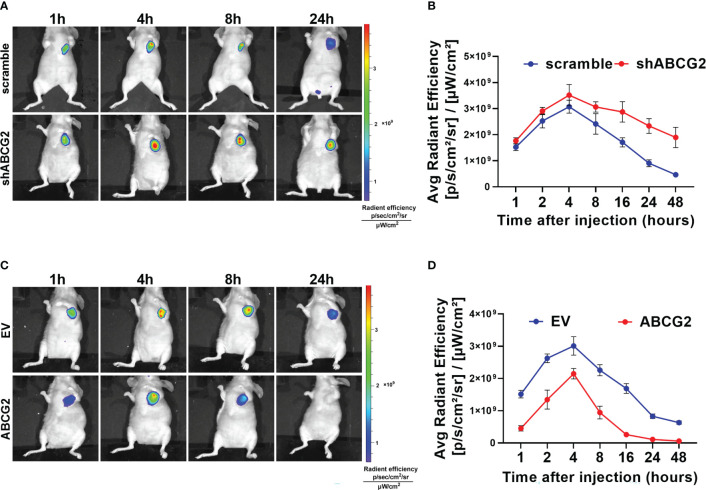
The efflux of MHI-148 from HCC cells is mainly mediated by ABCG2 *in vivo*. **(A)** Time-dependent intensity images of nude mice bearing subcutaneous tumor xenografts of SNU-739 cells with ABCG2-knockdown (shABCG2) after receiving an intravenous injection of MHI-148 (0.75 μmol kg^-1^). **(B)** Quantification of MHI-148 fluorescence intensity in **(A)**. **(C)** Time-dependent intensity images of nude mice bearing subcutaneous tumor xenografts of SNU-739 cells with overexpressed ABCG2 (ABCG2) after receiving an intravenous injection of MHI-148 (0.75 μmol kg^-1^). **(D)** Quantification of MHI-148 fluorescence intensity in **(C)**. Data shown are the mean ± SD from three independent experiments, where appropriate.

Furthermore, we explored the effect of ABCG2 on MHI-148 efflux in tumor-bearing mice. Our results showed that ABCG2 knockdown significantly prolonged the fluorescence signal duration of MHI-148 *in vivo*. The average radient efficiency of ABCG2 knockdown and control after injection 4h respectively is 3.5 ± 0.4×10^9^ and 3.0 ± 0.24×10^9^ [p/s/cm^2^/sr]/[μW/cm^2^]. (**Figures 5A, B**). In contrast, overexpression of ABCG2 reduced the fluorescence intensity of MHI-148 in tumors by 30% than control (The average radient efficiency after injection 4h respectively is 2.1 ± 0.16×10^9^ and 3.0 ± 0.3×10^9^ [p/s/cm^2^/sr]/[μW/cm^2^]) and the specific signal decayed rapidly within 8 hours after injection (**Figures 5C, D**).

These data collectively demonstrate that ABCG2 is involved in efflux of MHI-148 from HCC cells. We also found that low expression of ABCG2 leads to longer storage of MHI-148 in HCC cells, suggesting that ABCG2 may be another crucial regulator in clinical application of MHI-148.

### 3.4 β-catenin signaling pathway is involved in regulation of OATP2B1 and ABCG2 in HCC cells

**Figure 6:**
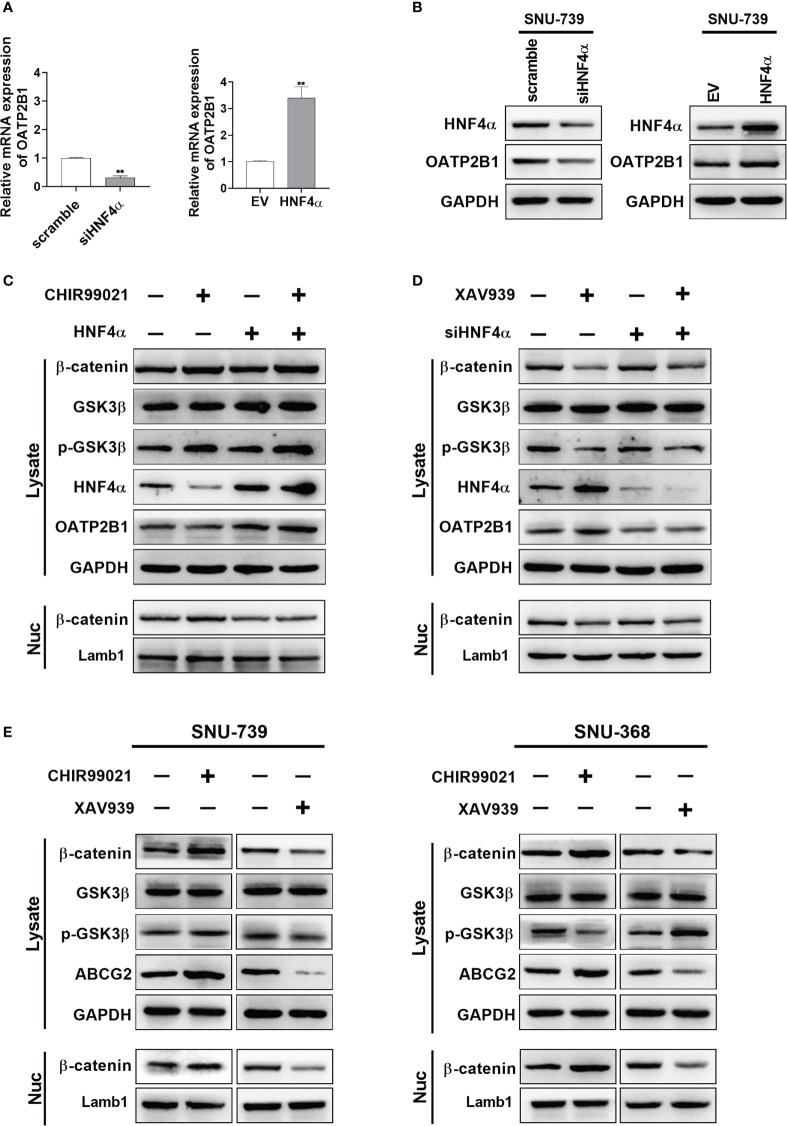
β-catenin signaling pathway is involved in regulation of OATP2B1 and ABCG2 in HCC cells. **(A, B)** QRT-PCR and western blotting analyses of OATP2B1 expression in SNU-739 cells with treatment as indicated. **(C, D)** Western blot analysis for β-catenin, GSK3β, p-GSK3β, HNF4α, and OATP2B1 in HCC cells with a panel of treatment as indicated. XAV939: cells were treated with XAV939 (5 μM) for 48 hours. CHIR99021: cells were treated with XAV939 (5 μM) for 48 hours. **(E)** Western blotting analysis for β-catenin, GSK3β, p-GSK3β, HNF4α, and ABCG2 in HCC cells with treatment as indicated. XAV939: cells were treated with XAV939 (5 μM) for 48 hours. CHIR99021: cells were treated with XAV939 (5 μM) for 48 hours. Data shown are the mean ± SD from three independent experiments, where appropriate. **P < 0.01.

A series of previous studies have showed that Wnt/β-catenin signaling regulates the expression of hepatocyte nuclear factor 4α (HNF4α), which binds to the proximal region of the promoter of *OATP2B1* gene to regulate the expression of OATP2B1. Therefore, we addressed whether β-catenin signaling regulates the uptake of MHI-148 by inducing the expression of OATP2B1 through HNF4α in HCC cells. As shown in **Figures 6A, B** and **Supplementary Figure 5A**, **4B**, HNF4α knockdown significantly decreased the mRNA and protein levels of OATP2B1 in SNU-739 cells. In contrast, HNF4α overexpression exhibited an opposite effect.

Then, we found that XAV-939, a β-catenin inhibitor which promotes β-catenin ubiquitination, induced the expression of HNF4α and OATP2B1. Whereas knockdown of HNF4α suppressed the expression of OATP2B1 in HCC cells (**Figure 6C**). In addition, as expected, the treatment with CHIR99021 which functions as an activator of β-catenin signaling pathway clearly decreased the expression of HNF4α and OATP2B1, and overexpression of HNF4α restored the protein level of OATP2B1 in HCC cells (**Figure 6D**). A concentration of 5μM was selected for two reagents to well regulate β-catenin (**Supplementary Figures 5C**, **4D**). As the expression of β-actin could be affected by intervention of β-catenin signaling pathway, GAPDH was used as the internal reference to replace β-actin.

After 48 hours treatment of the two reagents, HNF4α expression showed an obvious reduction in β-catenin-activated cells and a marked induction in β-catenin-inhibited cells (**Figure 6E**). The β-catenin has also been reported to be a transcriptional activator of ABCG2. We examined the effect of CHIR99021 and XAV-939 on ABCG2 expression in HCC cells. As shown in **Figure 6E**, CHIR99021 activated β-catenin signaling pathway and increased the protein level of ABCG2. In addition, XAV-939 remarkably suppressed β-catenin signaling and reduced expression of ABCG2.

### 3.5 The intensity of Near-Infrared fluorescence imaging is enhanced by inhibiting β-catenin signaling pathway

**Figure 7:**
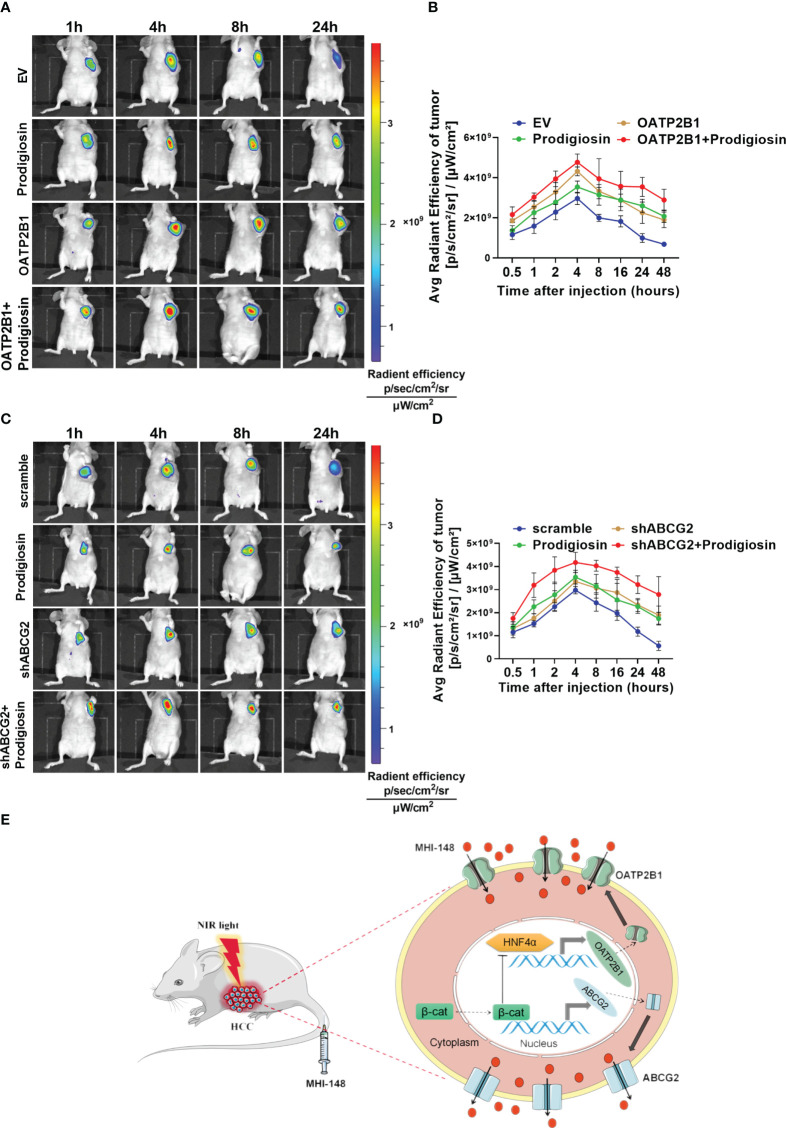
The intensity of NIRF imaging is enhanced by inhibiting the β-catenin signaling pathway. **(A, C)** Time-dependent intensity images of nude mice bearing subcutaneous tumors xenografts of SNU-739 cells treated as indicated after receiving an intravenous injection of MHI-148 (0.75 μmol kg-1). Prodigiosin (5 mg kg-1) was injected twice weekly for 1 week after the tumor volume reached approximately 50 mm^3^. **(B, D)** Quantification of MHI-148 fluorescence intensity in **(A, C)**. Data are presented as the ratio of intensity of dye accumulation as normalized to that of blank region (n = 5, mean ± SD). **(E)** Schematic depicting the underlying molecular mechanisms of membrane transporters-mediated tumor-specific accumulation and retention of MHI-148 in HCC cells.

To further investigate the regulatory function of β-catenin signaling pathway in MHI-148 imaging in HCC cells, we established the nude mice model. We found that not only the peak fluorescence signal was increased after injection especially in OATP2B1 overexpression group than control (The average radient efficiency after injection 4h respectively is 4.8 ± 0.4×10^9^ and 4.3 ± 0.2×10^9^ [p/s/cm^2^/sr]/[μW/cm^2^]), but also the fluorescence intensity was decreased more slowly when compared with tumors without treatment of a β-catenin inhibitor of Prodigiosin (**Figures 7A, B**). Meanwhile, the treatment with Prodigiosin significantly improved the intensity of MHI-148 imaging in ABCG2 knockdown group and extended the work time of the dye than control (The average radient efficiency after injection 4h respectively is 4.2 ± 0.4×10^9^ and 3.3 ± 0.3×10^9^ [p/s/cm^2^/sr]/[μW/cm^2^]) (**Figures 7C, D**).

Collectively, our data demonstrate that transporters of OATP2B1 and ABCG2, which regulated by β-catenin signaling pathway, mediate tumor-specific retention of MHI-148, and the inhibition of β-catenin signal pathway enhances the accumulation of MHI-148 in HCC tissues, which improves the efficacy of tumor imaging with MHI-148 *in vivo* (**Figure 7E**).

## 4 Discussion

The reduction of normal structural damage and removal of all malignant tissue is vital for HCC patients during hepatic resection, so precise indication of the target tissue is an important indicator for the clinical application of NIRF. Although ICG has so far been the only available NIR fluorophores approved by the US FDA for regular surgical treatments including the imaging of HCC, studies have shown that it does not discriminate well between benign and malignant tissues of the liver. Takemura et al. have pointed that the limitation of ICG fluorescent imaging mainly includes whole liver staining because ICG is absorbed by hepatocytes (30). HCC cells in poorly differentiated or metastatic liver cancer tissues hardly take up ICG, which influence the identification of HCC tissues by ICG (31). Gotoh et al. have reported that ICG is not able to distinguish benign and malignant tumors well, and its accumulation in adjacent non-tumor liver tissues leads to a false positive rate of 40–50% during detection of HCC (6, 9). In the present study, we identified the aggregation of ICG predominantly in normal liver epithelial cells rather than HCC cells both *in vitro* and *in vivo*. This finding suggests that more evidence is needed to clarify the role of NIRF imaging in HCC surgery, and the underlying mechanisms which involved in the absorption, distribution, and efflux of NIRF probe in HCC cells is crucial.

Different mechanisms of targeting tumor suggest that MHI 148 has more advantages in HCC imaging. A conventional approach to tumor specific imaging utilizing NIR fluorophores including ICG is to design targeted delivery modification, mostly by chemical conjugation to tumor-specific ligands (14–17). However, the limitation of these approaches leads to a narrow spectrum of tumor probes, as they only detect specific types of cancer cells with well-characterized surface properties, whereas tumors are notorious for their heterogeneity. In addition, the specificity and affinity of these tumor targeting probes vary according to chemical or physical conditions (32). Therefore, new molecular probes with high sensitivity for accurate tumor detection are needed (33). Unlike the ICG that needs targeted modification for tumor imaging, MHI-148 is spontaneously taken up and specifically accumulated in cancer cells, thus providing the advantage of tumor-specific imaging (18). Chung et al. have demonstrated that MHI-148 is retained in various kinds of cancer cells, tumor xenografts, and spontaneous tumors in transgenic mice (34). They have also found that MHI-148 can be used to detect cancer metastasis and cancer cells in blood with a high sensitivity (19). However, the mechanism by which MHI-148 is specifically taken up by malignant cells remains unclear and to be elucidated. In this study, we compared the specificity of tumor aggregation of MHI-148 and ICG *in vitro* and *in vivo* without chemical binding, and demonstrates that MHI 148 has liver cancer specific uptake *via* a mechanism in which the transporter for MHI-148 is highly expressed by liver cancer cells.

Tumor specific uptake and retention of MHI-148 may depend on its tumor specific expressed transporter. Since a group of SLC transporters called OATP family are involved in the transport of organic dyes into cells (22, 35), MHI-148 uptake may be mediated by transmembrane proteins of the OATP family. Previous studies have showed that the subtypes and expression levels of OATPs are different between cancer and normal cells. Certain members of OATPs are overexpressed in various human cancer tissues as well as in cancer cell lines (36). Therefore, the confirmation of OATPs as the key mediator of MHI-148 uptake and retention in tumor cells warrants further investigation. In our study, we firstly found that OATP2B1 was up-regulated in HCC tissues when compared with paired non-tumor tissues. OATP2B1 knockdown significantly reduced the uptake of MHI-148 in HCC cells. Although ICG has also been reported to be transported by OATP1B3, the broad expression profile of OATP1B3 in liver epithelial cells determines that ICG does not possess tumor-specific uptake in HCC cells.

In general, the uptake transporters of SLCs and efflux transporters of ABCs often share similar tissue distributions and overlapped substrate specificities, coordinating with each other for the transport of a wide array of substrates. Consequently, not only the changed expression level of SLCs influences the cellular uptake of exogenous substances, but also the dysregulation of ABCs critically affects the retention of substances in cells. It has been demonstrated that the activity of ABC transporters, which cause the efflux of chemotherapeutic agents and leads to decreased intracellular drug accumulation in various cancers, is associated with drug resistance (37). Previous studies have reported that immature hepatocytes often have impaired expression of ABC transporters, which are essential for the transport of many organic anions, including NIRF dyes (38–40). In addition, Kokudo et al. have proposed that the ICG accumulated in well-differentiated HCC cells may be related to ABC transporters anomalies, which lead to intracellular accumulation of ICG (41). These results suggest that ABC transporters may play a key role in tumor-specific retention of NIH-148 in HCC cells. Our current study for the first time demonstrated that the ABCG2 was involved in the efflux of MHI-148 from HCC cells. The expression of ABCG2 was down-regulated in HCC tissues when compared with paired non-tumor tissues, and knockdown of ABCG2 further prolonged the storage time of MHI-148 in HCC cells.

The precise mechanism by which the expression of OATP2B1 is regulated, however, remains elusive. Multiple transcription factors have been shown to play a potential role, but a definitive answer is still lacking especially in HCC cells (42). Knauer et al. have observed that HNF4α is involved in the regulation of hepatic OATP2B1 expression, indicating that knockdown of HNF4α results in a significant decrease in OATP2B1 transcription (43). Consistently, we demonstrated that the increased OATP2B1 expression was induced by up-regulated HNF4α. Previous studies have reported that the increase of HNF4α expression is associated with HCC progression (44, 45), and β-catenin signaling plays an important role in regulating HNF4α (46). Youmna Ali et al. have shown that Wnt inhibitors selectively modulate the function of multiple organic anions transporters (47). It has also been reported that the expression level of OATPs and ABCs is strongly associated with Wnt/β-catenin signaling (48, 49). All these studies strongly support our finding that β-catenin signaling pathway is involved in regulation of OATP2B1 and ABCG2 in HCC cells.

In summary, our data demonstrate that NIRF dye MHI-148 is practicable for effective imaging with high specificity in HCC cell. Moreover, our study provides the supporting evidence that the membrane transporters of OATP2B1 and ABCG2 regulated by β-catenin signaling pathway mediate tumor-specific accumulation and retention of MHI-148 in HCC cell. All these findings indicate that the distribution and expression level of transporters determines tissue specificity and imaging efficacy of NIRF dyes, suggesting that strategy targeting key components of MHI-148 transport machinery may be a potential approach in improving clinical HCC imaging.

## 5 Conclusion

Our study uncovers a mechanism that links the distribution and expression of the membrane transporters OATP2B1 and ABCG2 to the tumor-specific accumulation of MHI-148, and provides evidence supporting a regulating role of the β-catenin signaling pathway in OATP2B1 and ABCG2- induced retention of MHI-148 in HCC tissues.

## Data availability statement

The original contributions presented in the study are included in the article/**Supplementary Material**. Further inquiries can be directed to the corresponding authors.

## Ethics statement

The studies involving human participants were reviewed and approved by the Ethics Committee of the Fourth Military Medical University. The patients/participants provided their written informed consent to participate in this study. The animal study was reviewed and approved by animal welfare ethics committee of the Fourth Military Medical University.

## Author contributions

Conception, design, interpretation, and manuscript writing and revision: JS and TR. Data acquisition, statistical and computational analysis, and technical support: JS, TR, YD, HG, GW, YG, MB, XD, ZZ, JA. Study supervision: JA and TR. All authors contributed to the article and approved the submitted version.
