# Supplementary material for: Near-infrared fluorescence imaging of hepatocellular carcinoma cells regulated by β-catenin signaling pathway
Source: Front Oncol. 2023 Mar 29;13:1140256. doi: 10.3389/fonc.2023.1140256 (PMC10090467; doi:10.3389/fonc.2023.1140256)
Supplement: Supplementary file 1 [file DataSheet_1.docx]

Supplementary Material

Near-Infrared Fluorescence Imaging of hepatocellular carcinoma cells regulated by β-catenin signaling pathway

Jian Song^1†^, Tingting Ren^2†*^, Yanheng Duan^1^, Haitao Guo^3^, Gang Wang^3^, Yu Gan^1^, Mengcai Bai^2^, Xiaotian Dong^1^, Zheng Zhao^4*^, Jiaze An^1*^

*** Correspondence:**Corresponding Author: Jiaze An
E-mail: anchen@fmmu.edu.cn

Tingting Ren
E-mail: [rtt419@fmmu.edu.cn](mailto:rtt419@fmmu.edu.cn)

Zheng Zhao
E-mail: zhaozheng710061@163.com

# Supplementary Figures and Tables

## Supplementary Figures

**Supplementary Figure 1**(A) the chemical structure of ICG. (B) the chemical structure of MHI-148.


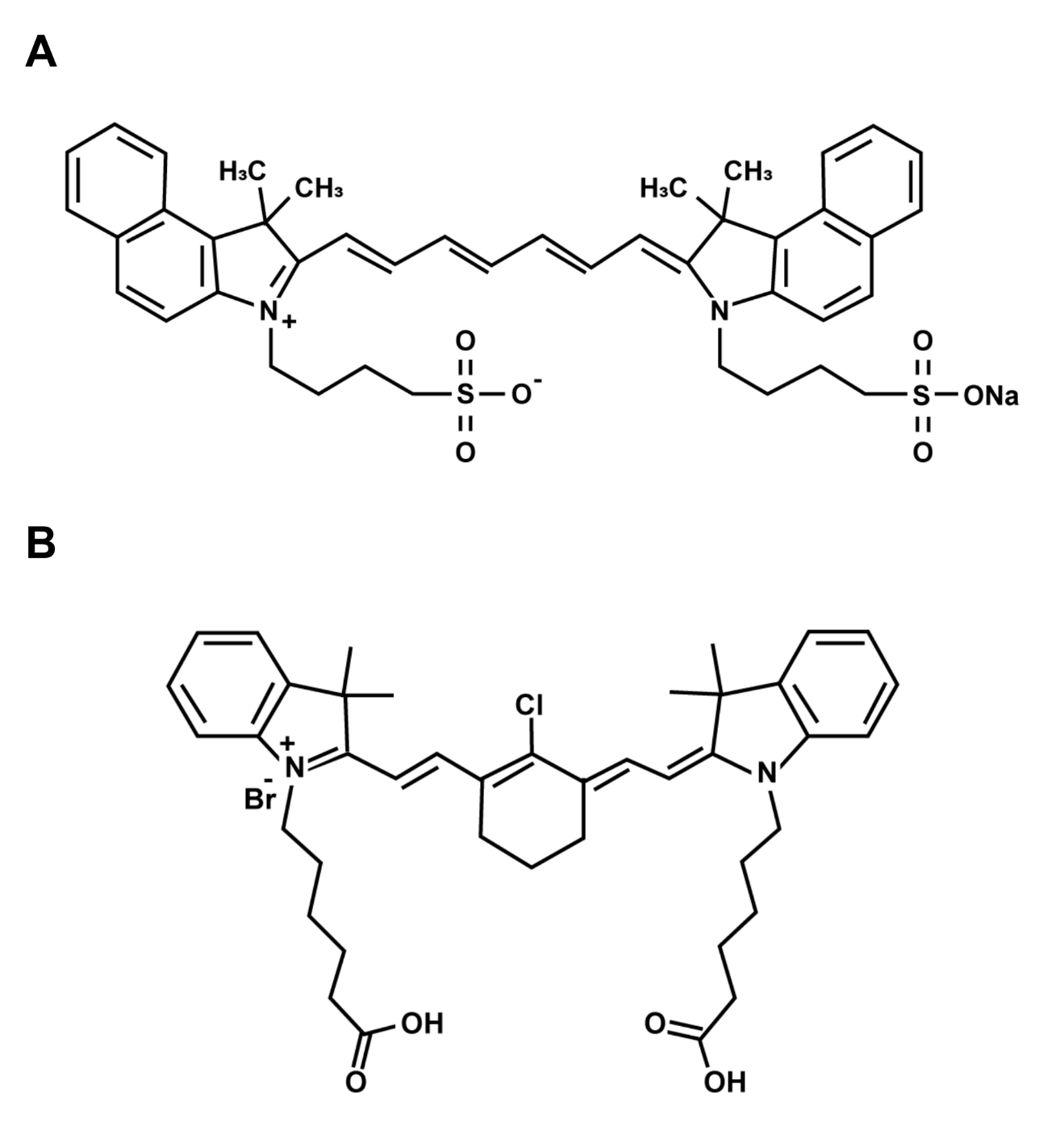


**Supplementary Figure 2.** (A-B) The optimal concentration of NIRF dyes in vitro was determined to be 10 μM. SNU-739 cells were treated with different concentrations of NIRF dyes for different time as indicated (excitation/emission, 750-800/820-860 nm: exposure time, 30 s) (A). MTS assay for cell viability in SNU-739 cells with treatments as indicated (B). (C) Representative images of cell staining with NIRF dyes in SNU-368. Cells were treated with 10μM MHI-148 or ICG for different time as indicated (excitation/emission, 750-800/820-860 nm: exposure time, 30 s). Scale bar, 20 μm. (D-G) In vivo biosafety evaluation of NIRF dyes. The mice were weighed at various time points after injection with NIRF dyes at a series of dosages as indicated (D). The mice were weighed at various time points after injection with NIRF dyes at a dosage of 0.75 μmol kg^-1^, and it showed that there was no significant difference between groups (E). Hepatic function markers (ALT, alanine aminotransferase; AST, aspartate aminotransferase; ALB, alanine albumin; TBil, total bilirubin), renal function markers (CREA, creatinine) and a heart function marker (CK, Creatine Kinase) were tested and found to be normal compared with the control group after 7 and 30 d, respectively, indicating no noticeable hepatic and renal dysfunctions induced by the NIRF dyes (F). Haematoxylin and eosin-stained tissues indicated that structural patterns of major organs harvested on day 30 from mice in the group of NIRF dyes injection were similar to those of the control group, with no signs of damage or other symptoms. Scale bar, 100 μm (G).


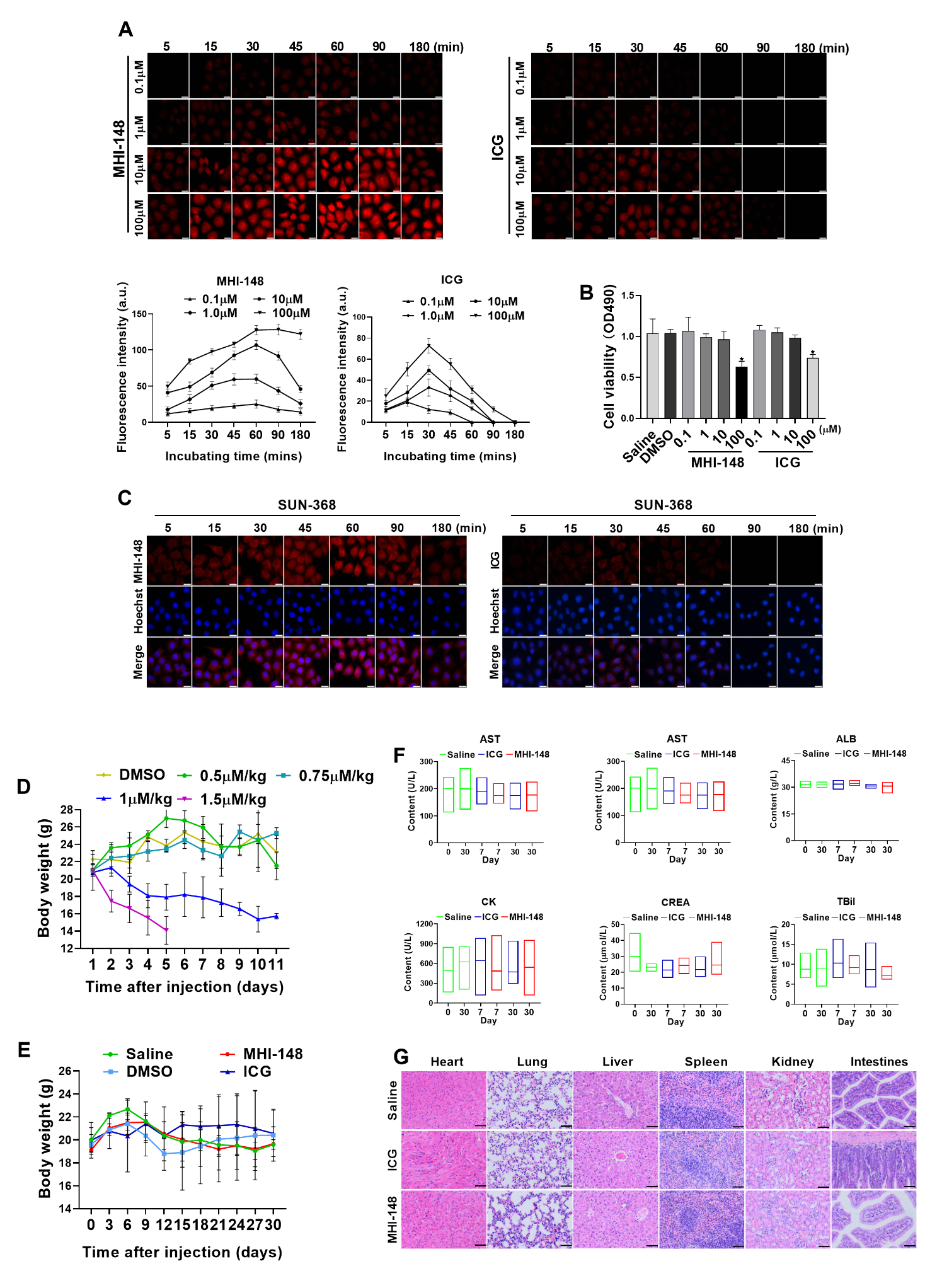


**Supplementary Figure 3.** (A) Western blot analyses of SLC transports protein level in SNU-739 cells transiently transfected with targeting siRNAs as indicated. (B) Western blot analysis of OATP2B1 protein level in HCC cell lines as indicated. (C) Western blot analysis of OATP2B1 protein level in HCC cells stably transfected with shRNA or force-expression vector of OATP2B1 as indicated. (shOATP2B1, shRNA against OATP2B1; scramble, control shRNA; OATP2B1, expression vector encoding OATP2B1; EV, empty vector). (D-E) Determination of MHI-148 uptake in SNU-368 cells with knockdown or overexpression of OATP2B1(excitation/emission, 750-800/820-860 nm: exposure time, 30 s). Data shown are the mean ± SD from three independent experiments, where appropriate.

**
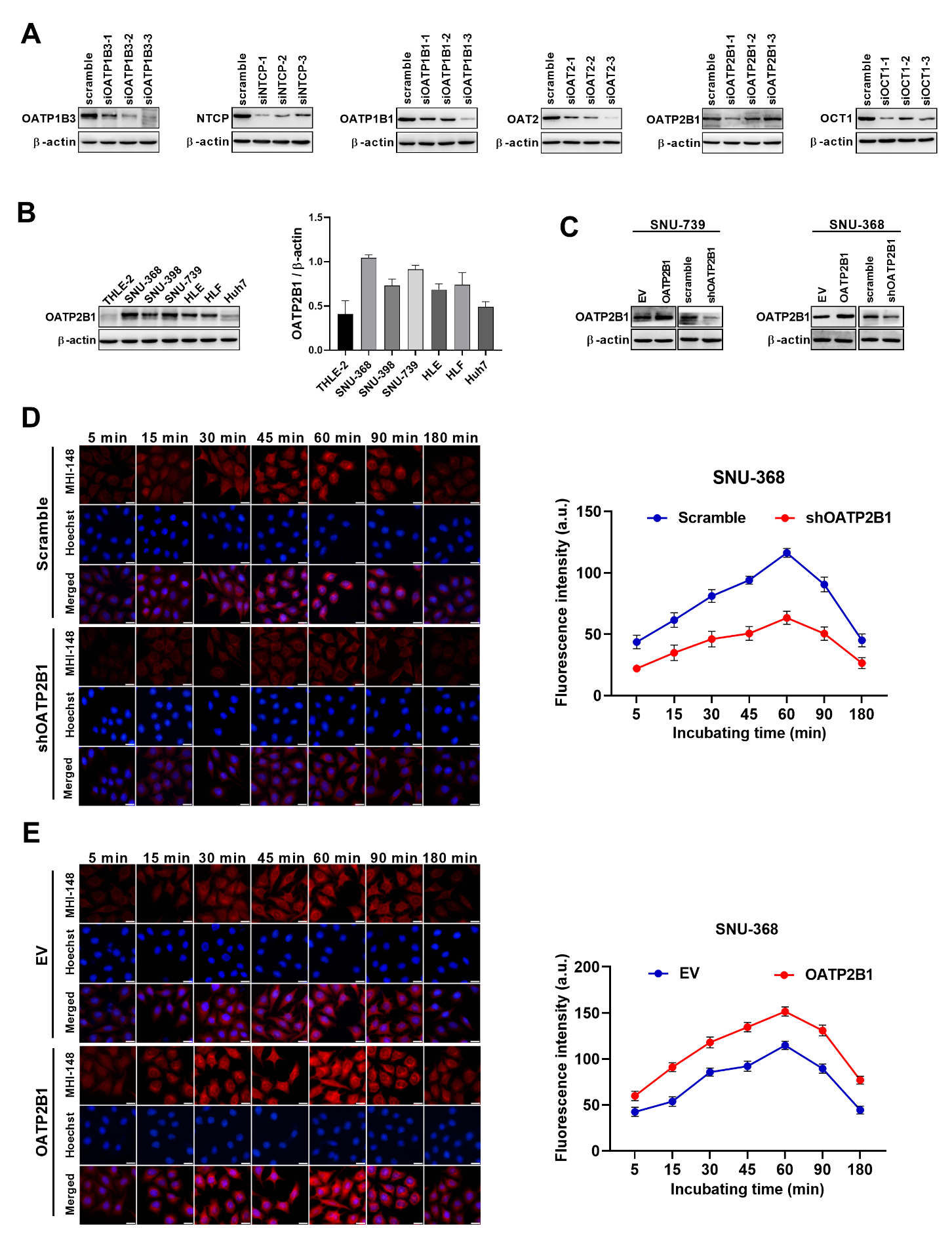
**

**Supplementary Figure 4.** (A) Western blot analyses of ABC transports protein level in SNU-739 cells transiently transfected with targeting siRNAs as indicated. (B) Western blot analysis of ABCG2 protein level in HCC cell lines as indicated. (C) Western blot analysis of ABCG2 protein level in HCC cells stably transfected with shRNA or force-expression vector of ABCG2 as indicated. (shABCG2, shRNA against ABCG2; scramble, control shRNA; ABCG2, expression vector encoding ABCG2; EV, empty vector). (D-E) Determination of MHI-148 retention in SNU-368 cells with knockdown or overexpression of ABCG2. Data shown are the mean ± SD from three independent experiments, where appropriate(excitation/emission, 750-800/820-860 nm: exposure time, 30 s).


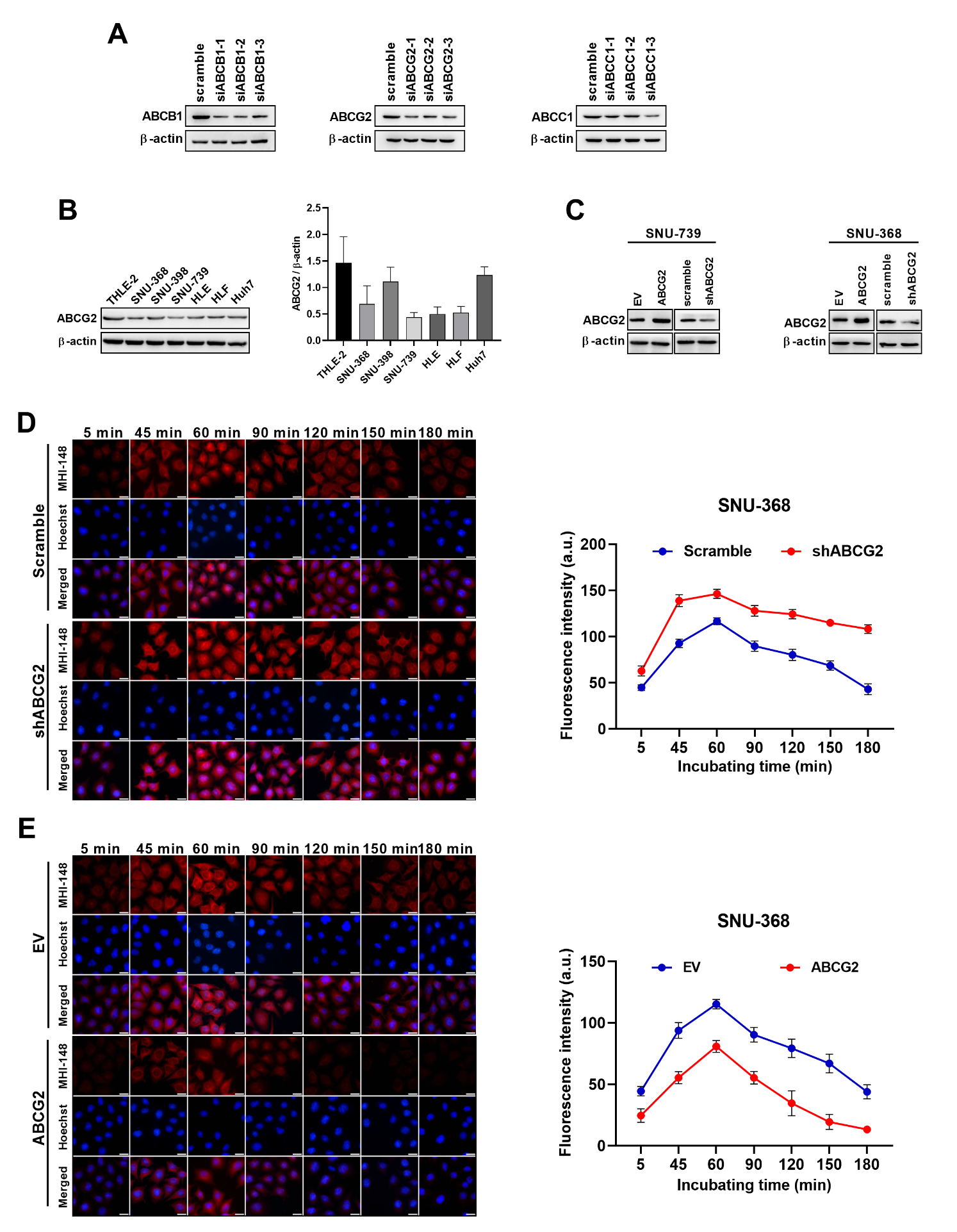


**Supplementary Figure 5.** (A) and (B) Western blot analysis of HNF4α protein level in HCC cells transfected with siRNA or force-expression vector of HNF4α as indicated. (C) and (D) Western blot analysis of the protein levels of β-catenin and HNF4α in SNU-739 cells treated with 5 μM CHIR99021 and XAV939, respectively. HNF4α expression showed an obvious change after 48 hours incubation of CHIR99021 or XAV939.

**
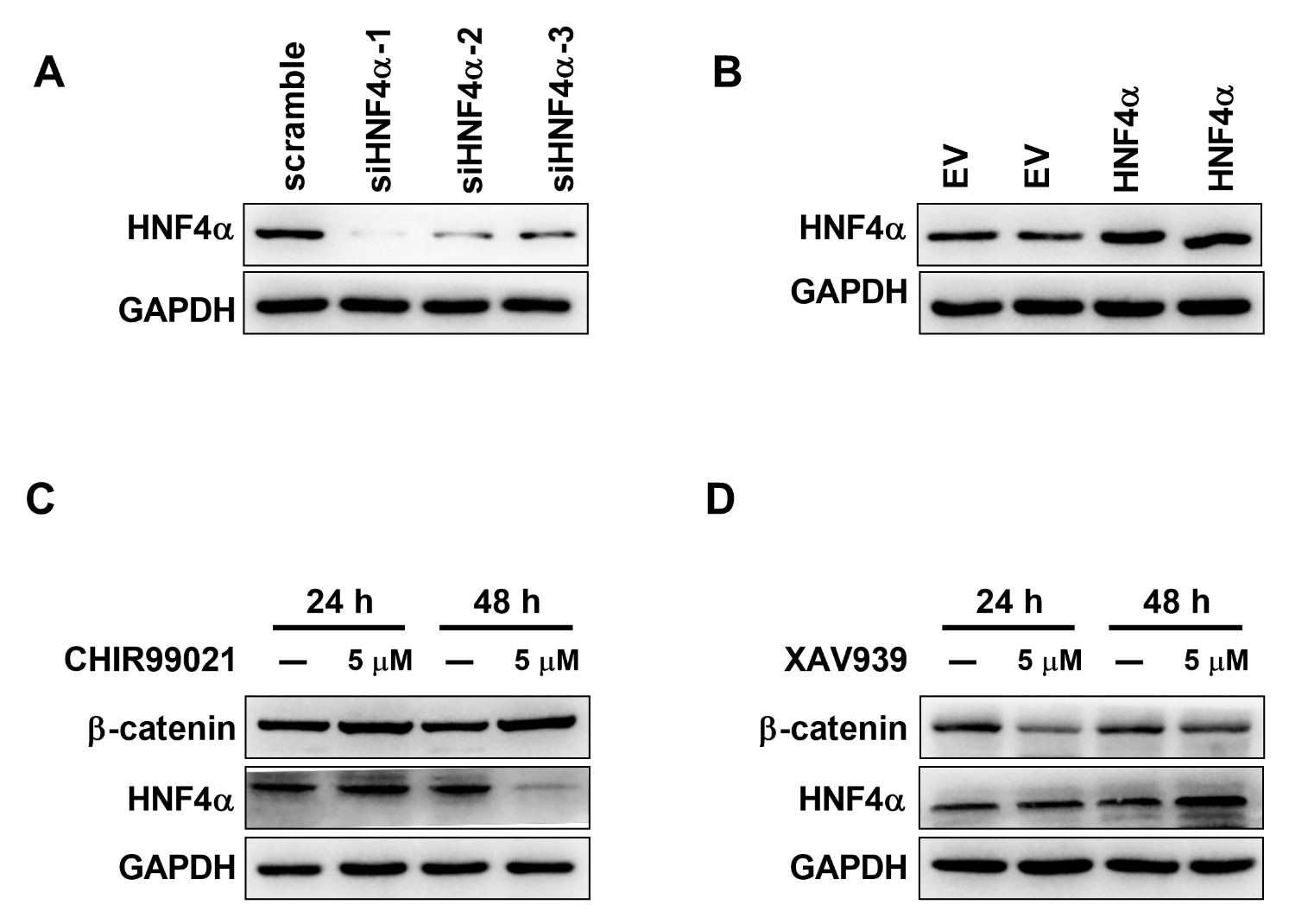
**

## Supplementary Tables

**Supplementary Table 1.** Primary antibodies used for western blot.

| **Antibody** | **Company (Cat.NO.)** | **Working dilutions** |
| --- | --- | --- |
| β–catenin | Proteintech (51067-2-AP) | WB: 1/1000 |
| ABCG2 | ABclonal (A17908) | WB:1/1000 |
| ABCG2 | CST (42078S) | WB:1/1000 |
| OATP2B1 | ABclonal (A10073) | WB:1/1000 |
| OATP2B1 | Abcam (ab203215) | WB:1/1000 |
| OATP1B3 | MERCK (HPA004943) | WB:1/1000 |
| OATP1B1 | ABclonal (A17082) | WB:1/1000 |
| OAT2 | ABclonal (A15137) | WB:1/1000 |
| OCT1 | ABclonal (A1682) | WB:1/1000 |
| ABCB1 | ABclonal (A11747) | WB:1/1000 |
| ABCC1 | ABclonal (A11153) | WB:1/1000 |
| HNF4α | ABclonal (A2085) | WB:1/1000 |
| Lamin B1 | Proteintech (12987-1-AP) | WB:1/1000 |
| GSK-3β | Proteintech (22104-1-AP) | WB:1/1000 |
| p-GSK-3β | ABclonal (AP0039) | WB:1/1000 |
| β–actin | TDY BIOTEC (TDY051C) | WB: 1/3000 |
| GAPDH | Proteintech (60004-1-Ig) | WB:1/1000 |
| β–catenin | Proteintech (51067-2-AP) | IHC:1/250 |
| ABCG2 | Abcam (ab3380) | IHC:1/200 |
| OATP2B1 | Abcam (ab222094) | IHC:1/200 |
| HNF4α | Affinity (DF6809) | IHC:1/100 |

**Supplementary Table 2.** Distribution of HCC patients’ characteristics.

| **Variable** | **All patients, n (%), n = 100** |
| --- | --- |
| Sex |  |
| Female | 23 |
| Male | 77 |
| Age, years |  |
| <52 | 44 |
| ≥52 | 56 (44.9%) |
| Serum HBsAg |  |
| Negative | 13 |
| Positive | 87 |
| Serum AFP, ng/ml |  |
| <200 | 39 |
| ≥200 | 61 |
| Size of tumor, cm |  |
| <5cm | 44 |
| ≥5cm | 56 |
| TNM stage |  |
| I+II | 36 |
| III+IV | 64 |

PVT, portal vein thrombus; TNM, tumor node metastasis; AFP, alphafetoprotein.

**Supplementary Table 3.** **Sequence of primers**

| **1. Primers used in qPCR analysis** | | |
| --- | --- | --- |
| HNF4α | forward primer | CCTACCTCAAAGCCATCAT |
|  | reverse primer | ATGTAGTCCTCCAAGCTCAC |
| OATP2B1 | forward primer | CTTCATCTCGGAGCCATACC |
|  | reverse primer | GCTTGAGCAGTTGCCATTG |
| **2.siRNA** |  |  |
| siABCB1-1 | sense | GAGCUUAACACCCGACUUAUU |
|  | antisense | AAUAAGUCGGGUGUUAAGCUC |
| siABCB1-2 | sense | UCCCGUAGAAACCUUACAUUUAUGG |
|  | antisense | CCAUAAAUGUAAGGUUUCUACGGGA |
| siABCB1-3 | sense | CCAGGUAUGCCUAUUAUUAUU |
|  | antisense | AAUAAUAAUAGGCAUACCUGG |
| siABCC1-1 | sense | CGGUCUAUUCCCAUUUCAACGAGA |
|  | antisense | UCUCGUUGAAAUGGGAAUAGACCG |
| siABCC1-2 | sense | GGAAGGGAGUUCAGUCUUCTT |
|  | antisense | GAAGACUGAACUCCCUUCCTC |
| siABCC1-3 | sense | GCAACUGCAUCGUUCUGU |
|  | antisense | ACAGAACGAUGCAGUUGC |
| siABCG2-1 | sense | GCAGAUGCCUUCUUCGUU |
|  | antisense | AACGAAGAAGGCAUCUGC |
| siABCG2-2 | sense | GCAGACUUCUUGGACAUCAUU |
|  | antisense | AAUGAUGUCCAAGAAGUCUGC |
| siABCG2-3 | sense | GCGGAUACUACAGAGUGUCAUCUU |
|  | antisense | AACAUGACACUCCGCAGUAUCCGC |
| siHNF4α-1 | sense | UGUGCAGGUGUUGACGAUGdTdT |
|  | antisense | CAUCGUCAACACCUGCACAdTdT |
| siHNF4α-2 | sense | GUGGAGAGUUCUUACGACAUUdTdT |
|  | antisense | UUCACCUCUCAAGAAUGCUGUdTdT |
|  |  |  |
| siHNF4α-3 | sense | CCAAUGUCAUUGUUGCUAAUU |
|  | antisense | AAUUAGCAACAAUGACAUUGG |
| siOATP2B1-1 | sense | GCGCCUUUAUGUGGACAUUAA |
|  | antisense | UUAAUGUCCACAUAAAGGCGC |
| siOATP2B1-2 | sense | AUGAUCUCCGGCUACCUAAAG |
|  | antisense | CUUUAGGUAGCCGGAGAUCAU |
| siOATP2B1-3 | sense | UGGUAUCAGCCUGACCAUAAA |
|  | antisense | UUUAUGGUCAGGCUGAUACCA |
| siOATP1B3-1 | sense | GAUAAUACUUGUACAAGGATT |
|  | antisense | UCCUUGUACAAGUAUUAUCTT |
| siOATP1B3-2 | sense | GUUGCAAUUCAAGUCAUAATT |
|  | antisense | UUAUGACUUGAAUUGCAACTT |
| siOATP1B3-3 | sense | GCCCUGUCAUUCAGCUAUATT |
|  | antisense | UAUAGCUGAAUGACAGGGCTT |
| siNTCP-1 | sense | AAGAUCUCAAUCUCGGGAAUC |
|  | antisense | GAUUCCCGAGAUUGAGAUCUU |
| siNTCP-2 | sense | AGGUCCCCUAGAAGAAGAAC |
|  | antisense | GUUCUUCUUCUAGGGGACCUG |
| siNTCP-3 | sense | AACACUUCCGGAAACUACUGU |
|  | antisense | ACAGUAGUUUCCGGAAGUGUU |
| siOATP1B1-1 | sense | CAGGAUAACUCCUACUGAUTT |
|  | antisense | AUCAGUAGGAGUUAUCCUGTT |
| siOATP1B1-2 | sense | GGUCAUACAUGUGGAUAUATT |
|  | antisense | UAUAUCCACAUGUAUGACCTT |
| siOATP1B1-3 | sense | GGGUCAUGUAGGACAUAUATT |
|  | antisense | UAUAUGUCCUACAUGACCCTT |
| siOCT1-1 | sense | CGAUUUACCUUAAGGUCCATT |
|  | antisense | UGGACCUUAAGGUAAAUCGTT |
| siOCT1-2 | sense | GCGGAGGAGCUGAACUAUATT |
|  | antisense | UAUAGUUCAGCUCCUCCGCTT |
| siOCT1-3 | sense | CGGUGGCUGUUAUCACAAATT |
|  | antisense | UUUGUGAUAACAGCCACCGTT |
| siOAT2-1 | sense | CUGCUAGUGUCCUCCGAUATT |
|  | antisense | UAUCGGAGGACACUAGCAGTT |
| siOAT2-2 | sense | GAGUGAACUUCUCCUAUUATT |
|  | antisense | UAAUAGGAGAAGUUCACUCTT |
| siOAT2-3 | sense | GAAGCAGGUCCAGAACUAATT |
|  | antisense | UUAGUUCUGGACCUGCUUCTT |
| **3. shRNA** |  |  |
| sh-OATP2B1 | 5’-CCGGGCGCCTTTATGTGGACATTAACTCGAGTTAATGTCCACATAAAGGCGCTTTTTT-3’ | |
| sh-ABCG2 | 5’-CCGGGCCTACCTGAAATTGTTATTTCAAGAGAATAACAATTTCAGGTAGGCTTTTTT-3’ | |
| scramble | 5’-CCGGATGATCTCCGGCTACCTAAAGCTCGAGCTTTAGGTAGCCGGAGATCATTTTTTTG-3’ | |
